# The Unusual Case of Fibroma of Tendon Sheath in a Young Girl with Turner Syndrome Undergoing Growth Hormone Treatment

**DOI:** 10.4274/jcrpe.galenos.2020.2019.0223

**Published:** 2021-02-26

**Authors:** Yong Hee Hong, Dong Gyu Kim, Jong Hyun Lee, Min Jung Jung, Chang Yong Choi

**Affiliations:** 1 Soonchunhyang University Bucheon Hospital, Soonchunhyang University College of Medicine, Department of Pediatrics, Bucheon, Republic of Korea; 2 Soonchunhyang University Bucheon Hospital, Soonchunhyang University College of Medicine, Department of Plastic and Reconstructive Surgery, Bucheon, Republic of Korea; 3 Soonchunhyang University Gumi Hospital, Soonchunhyang University College of Medicine, Department of Pediatrics, Gumi, Republic of Korea; 4 Soonchunhyang University Bucheon Hospital, Soonchunhyang University College of Medicine, Department of Pathology, Bucheon, Republic of Korea

**Keywords:** Fibroma, tendons, Turner syndrome, growth hormone

## Abstract

Fibroma of tendon sheath (FTS) is an uncommon mass that arises from the tendon sheath of extremities. The tumor typically affects adults between ages 20 and 50 years with a predominance in males. To date, growth hormone (GH) treatment is safe for children with Turner syndrome without risk factors and is accepted worldwide. This article reports the case of a nine-year-old female patient with Turner syndrome and FTS during GH treatment. She had been treated with daily subcutaneous GH to improve growth failure with a mean dose of 0.28 mg/kg/week and the level of insulin-like growth factor-1 was within the normal range. During the follow-up period, she complained about a mass in her hand, subsequently diagnosed as FTS. This report illustrates the clinical impact of Turner syndrome and GH treatments on the occurrence of this tumor through literature reviews. Further studies are needed to highlight the association between FTS and GH treatment, especially in Turner syndrome.

**What is already known on this topic?**

Fibroma of tendon sheath (FTS) is an uncommon mass that arises from the tendon sheath of extremities, particularly in children. Recombinant human growth hormone (rGH) treatment in patients with Turner syndrome is accepted worldwide because the syndromic short stature. The International Turner Syndrome Consensus Group does not recommend a specific cancer screening protocol.

**What this study adds?**

To our knowledge, there have been no reports of the co-occurrence of Turner syndrome and FTS in a young child during rGH treatment. The rGH treatment seems to affect the growth of tumor in this case because of early-onset and rapid growth compared with well-known characteristics of FTS in adults. When a hand mass occurs in Turner syndrome patients undergoing rGH treatment, it may be worth considering FTS as a possible diagnosis in order to not miss appropriate management.

## Introduction

Turner syndrome patients show increased morbidity due to metabolic disease, thyroid and particularly to the well-known cardiac and aortic dissection risk. The risk of cancer in Turner syndrome patients has also been studied. To date, recombinant human growth hormone (rGH) treatment in patients with Turner syndrome is accepted worldwide because of the syndromic short stature. Apart from the beneficial effect of GH on stature, childhood GH therapy in Turner syndrome favorably affects the cardiovascular system via improvement in the lipid profile and a decreased prevalence of arterial hypertension (1). The long‐term safety of GH was not associated with an increased risk of new malignancy, leukemia, non-leukemic extracranial tumors or recurrence of intracranial malignancy in patients without risk factors during rGH treatment (2). There was evidence of an increased risk of a second neoplasm in children previously treated for cancer. However, current experimental data supports the hypothesis that the GH/insulin-like growth factor-1 (IGF-1) status may facilitate carcinogenesis and influence cancer biology (3,4).

The first fibroma of tendon sheath (FTS) was described by Geschickter and Copeland (5) about 70 years ago. FTS is an uncommon, benign lesion arising from the tendon sheath of extremities, particularly the hands. The tumor typically affects adults between 20 and 50 years with a male to female ratio ranging from 1.5:1 to 3:1 (6). The clinical course of FTS usually occurs years after its formation as a slow-growing, dense, painless or mildly tender mass that is firmly attached to the tendon sheath.

In this case, the patient who had Turner syndrome had been treated with GH for years. Numerous case reports about FTS have been published. However, to our knowledge, there have been no reports of the co-occurrence of Turner syndrome and FTS in a young child during rGH treatment. We herein present a particular case of FTS in Turner syndrome, to emphasize its unusual clinical course through this case report and review of the literature.

## Case Report

**Figure 1:**
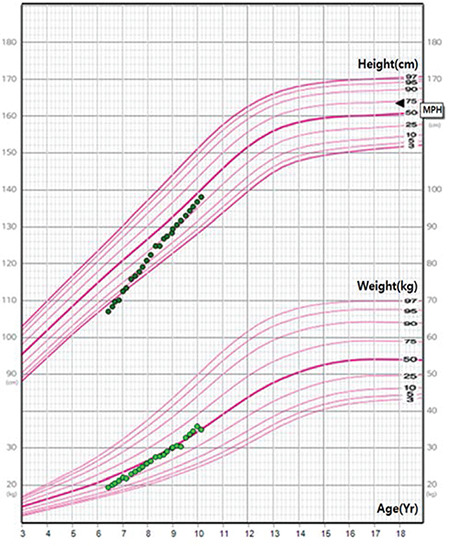
The patient’s growth curve during the follow-up period. The patient was diagnosed with Turner syndrome at the age of 6.3 years and underwent recombinant human growth hormone treatment.

The patient was followed up for Turner syndrome in the Department of Pediatrics. The karyotype was 45,X[22]/47,XXX[8] mosaicism. After the condition was diagnosed at the age of six years and four months, she was treated with daily subcutaneous rGH to improve growth failure with a mean dose of 0.28 mg/kg/week (Table 1). The height standard deviation score increased from -2.17 to -0.09 and her growth velocity increased from 4 to 5 cm/year before treatment to 7 to 8 cm/year during treatment (Figure 1). During rGH treatment, she did not have an elevated glucose level or abnormal thyroid function and the level of IGF-1 was within the normal range. During the follow-up period, she complained about a mass in her hand and was referred to the Department of Plastic and Reconstructive Surgery for the treatment of a nontender, relatively rapid-growing mass of two-months duration.

**Figure 2:**
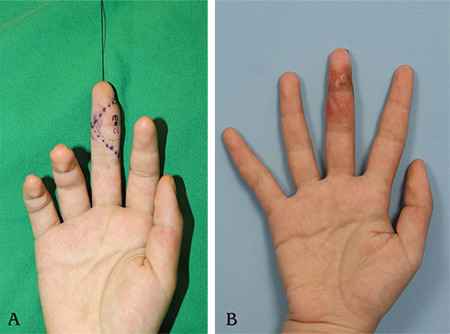
Appearance of the patient’s hand before and after surgery. A) Note the mass on the right middle finger distal interphalangeal joint. B) Postoperative appearance of the patient’s hand a month after the surgery.

**Figure 3:**
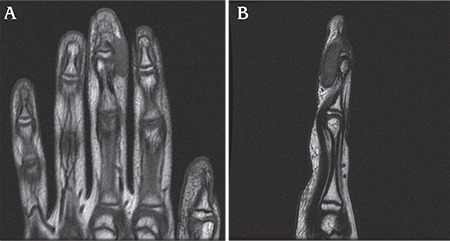
Preoperative magnetic resonance finding. A) Coronal fast spin-echo T1 image shows a mass of low signal intensity on the right middle finger distal interphalangeal joint. B) Sagittal fast spin echo T2 image shows a mass of equal-to-high signal intensity centrally with decreased signal peripherally.

The mass was located over the radial volar aspect of the right middle finger (Figure 2A). She had no neurological or vascular symptoms, and the range of movement of the right middle finger was not limited. She felt no discomfort but complained of the noticeable mass when flexing her finger. There was no history of previous penetrating or blunt trauma over the mass lesion. Sagittal fast spin-echo T2-weighted magnetic resonance image (MRI) revealed a mass with areas of iso-to-high signal intensity and dark signal foci at the periphery (Figure 3). Erosion of the adjacent bone was not seen, but attachment to the 3^rd^ flexor profundus tendon was marked. The neurovascular bundle was swept away laterally from the lesion. Originally, the first impression based on MRI was a giant cell tumor of tendon sheath.

**Figure 4:**
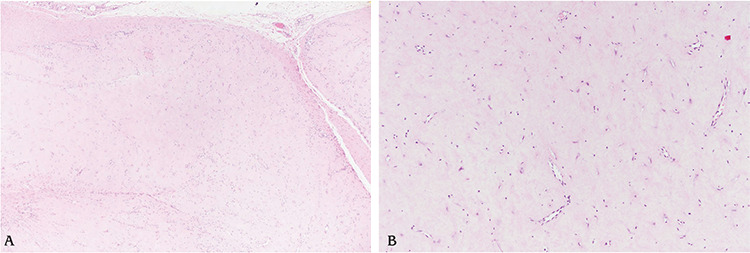
Histologic finding (hematoxylin and eosin stain). The tumor was a well-circumscribed nodule with clefts, attached to the flexor tendon (A, x40). It contained bland fibroblastic spindle cells in the dense collagenous stroma (B, x100).

The surgical procedure was performed under general anesthesia. Upon gross examination, the tumor appeared to be well-demarcated, lobulated, solid and oval-shaped. It was measured to be 2x1.8x1 cm, and its cut surface was white-tan and rubbery. Histopathologic examination confirmed the mass to be FTS (Figure 4).

As in our case, FTS can easily be confused with a giant cell tumor of tendon sheath, and a final accurate diagnosis is normally made by its histopathologic findings. Microscopically, a collagenous stroma and benign fibroblasts with low cellularity were noted. Histopathologic findings and results of immunohistochemistry were consistent with that of FTS. No early postoperative complications such as infection, bleeding, or dehiscence were noted. The patient achieved full range of motion of the affected finger with no pain or tenderness by one month after the surgery (Figure 2B).

## Discussion

The FTS in this present case developed in a patient with Turner syndrome. FTS is known to typically affect adults between 20 and 50 years with a predominance in males. In contrast, the patient in this case was female and only nine years old. Long-term studies have shown that early GH treatment can correct growth failure and normalize height in infants and children with Turner syndrome (7). Our patient had been treated with rGH for more than three years. Considering this unusual presentation and her special medial history, we supposed that the GH might affect the course of this patient. With this assumption, we investigated the clinical impact of Turner syndrome and GH treatment on the occurrence of this tumor through literature reviews.

Generally, it is accepted that the hormonal abnormalities and treatments for this syndrome might affect the risk of hormone-related cancers, and the chromosomal abnormality itself might affect cancer risk (8). Some large retrospective observational studies (8,9,10) have undertaken a comparison of cytogenetic and cancer registries data. They reported that the overall risk of cancer is possibly slightly raised (in one study only) (9) with standardized incidence ratios (SIR) between 0.9 and 1.34, but according to others (8,10), the overall risk of cancer was similar to that seen in the normal population. All reported that the incidence of breast cancer is reduced, the risk of melanoma increased between twofold and threefold, and the risk of nervous system malignancy increased between 4.3- and 6.6-fold with the SIR for meningioma increased between 12 and 14. Until prospective studies clarify the cost-effectiveness of routine screening, the International Turner Syndrome Consensus Group does not recommend a specific cancer screening protocol (11).

The final mediator of the growth promoting action of GH is IGF-1, which exerts potent anti-apoptotic and mitogenic activity in all cells and is expressed and secreted from many different types of cancer cells (12). There is considerable concern that GH treatment may be associated with tumor development. The potential relationship between GH treatment and increased risk of tumor development has been the subject of many studies. Although it is a reasonable assumption that there might be carcinogenic effects, still there is no evidence that GH treatment in young patients with growth disorders actually results in an increased risk of developing cancer relative to that expected in the normal population (13). Additionally, a recent, large, cross-European cohort study (14) also showed no clear, raised cancer risk in patients with growth failure without other major disease. The two aforementioned studies do not generally support a carcinogenic effect of rGH, but currently available experimental data does support the hypothesis that the GH/IGF-1 status may facilitate carcinogenesis (4).

The etiology of FTS is not certain, as it may represent a reactive fibrosing disease or a true neoplasm. However, after a (2;11) translocation was found by Dal Cin et al (15), it is now generally accepted that FTS is neoplastic. The presence of clonal chromosomal changes suggests a true neoplastic nature. Given the neoplastic nature of this lesion, a causal relationship between FTS and rGH treatment seems possible. This younger age of onset and relatively rapid growth also suggests a possibility that tumor growth might be affected by rGH treatment.

FTS is a benign process but may impose problematic anatomical and neurological complications if not treated promptly. About one-third of cases have been affected by neurologic symptoms due to compression (6). FTS has also been reported to cause a “trigger wrist” or limited flexion of fingers by adherence to the tendon (16). Therefore, in addition to timely management, early suspicion is also of great importance to avoid potential complications.

In conclusion, it was not clear that the rGH treatment facilitated the occurrence of FTS in a girl with Turner syndrome, but it seems possible because of the unusually young age of onset. The rGH treatment seems to affect the growth of the tumor in this case because of rapid growth compared with well-known characteristics of FTS in adults. To our knowledge, this is the first case of new-onset malignancy in a rGH-treated patient with Turner syndrome and without prior risk factors. If a hand mass occurs in Turner syndrome patients undergoing GH treatment, it may be worth considering FTS as a possible diagnosis in order to not miss appropriate management. For the present, decisive evidence should be explored to determine whether the relationship between rGH therapy and FTS occurrence is causal. Long-term clinical follow-up and further studies are required to highlight the link between FTS and GH treatment.

## Figures and Tables

**Table 1:**
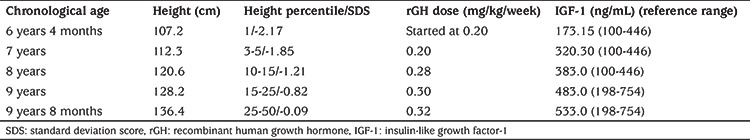
Growth data, recombinant human growth hormone dose and serum insulin-like growth factor-1 levels in our patient
